# Experimental Investigation of the Flexural Performance of Continuous Self-Compacting Concrete Beams with Natural and Recycled Aggregates

**DOI:** 10.3390/ma19020264

**Published:** 2026-01-08

**Authors:** Žarko Petrović, Bojan Milošević, Marija Spasojević Šurdilović, Andrija Zorić, Dragana Turnić

**Affiliations:** 1 Faculty of Civil Engineering and Architecture, University of Niš, 18000 Niš, Serbia; 2 Faculty of Mechanical Engineering and Civil Engineering in Kraljevo, University of Kragujevac, 36000 Kraljevo, Serbia

**Keywords:** self-compacting concrete, natural aggregates, recycled concrete aggregate, continuous reinforced concrete beams, flexural performance, sustainable concrete

## Abstract

This paper presents an experimental investigation on the flexural performance of continuous two-span reinforced concrete beams made with self-compacting concrete (SCC) incorporating natural and recycled coarse aggregates. A total of nine beams were tested under static loading conditions. The beams were divided into three groups based on different reinforcement ratios, and within each group, three aggregate replacement levels were used: 0%, 50%, and 100% recycled coarse aggregate. All beams were designed with identical cross-sections and subjected to two-point loading to simulate continuous support conditions. The study focused on evaluating cracking behavior, load–deflection response, and failure modes. The experimental results highlight that partial replacement with recycled aggregates (RAC50) can achieve comparable or even improved mechanical performance compared to natural aggregate beams, including enhanced compressive strength and ductility. Beams with 100% recycled aggregates (RAC100) showed slightly higher deflections and earlier crack initiation, particularly at lower reinforcement ratios, although overall flexural behavior remained consistent with natural aggregate concrete (NAC) beams. It was also observed that as reinforcement ratio increases, the influence of aggregate type diminishes, indicating that steel reinforcement predominantly governs the structural response at higher ratios. Crack widths and propagation patterns were systematically monitored, confirming that RAC beams maintain acceptable deformation and ductility under load. These findings emphasize the feasibility of using high-quality recycled aggregates in structural SCC elements, providing a sustainable alternative without compromising performance, and offering guidance for the design of continuous RAC beams.

## 1. Introduction

The global construction industry is facing a critical challenge: balancing infrastructure growth with environmental sustainability. One of the most pressing concerns is the depletion of natural resources, particularly natural aggregates, driven by the rising demand for concrete in urbanization and infrastructure development. Simultaneously, construction and demolition waste (C&DW) continues to accumulate at alarming rates, exacerbating landfill congestion and environmental degradation. As a result, the use of recycled concrete aggregate (RCA) has emerged as a sustainable alternative to natural coarse aggregates, offering both ecological and economic advantages by promoting material reuse and reducing resource extraction.

Parallel to this development is the increasing adoption of self-compacting concrete (SCC), a highly flowable, non-segregating concrete mix that can fill complex formwork and densely reinforced sections without mechanical vibration. Originally developed in Japan during the 1980s to overcome issues related to inadequate compaction, SCC enhances construction efficiency and improves surface finishes, while also reducing labor and equipment needs [1,2,3,4]. The synergistic integration of RCA into SCC presents a promising solution for sustainable construction; however, it introduces unique technical challenges due to the intrinsic characteristics of recycled aggregates, such as higher water absorption, lower density, and the presence of adhered mortar [5,6,7,8,9].

Studies have shown that increasing RCA content in SCC generally leads to reduced workability, lower slump flow, increased viscosity, and reduced passing ability due to higher internal friction [2,10,11]. Kou and Poon [2] observed increased T500 flow times with higher RCA content, while Grdić et al. [3] demonstrated that SCC with up to 50% RCA replacement can maintain satisfactory fresh properties when high-range water-reducing admixtures and pre-saturated aggregates are used. With appropriate mix design strategies—including adjustment of mixing water, increased paste volume, and the use of supplementary cementitious materials (SCMs) such as fly ash, silica fume, or metakaolin—SCC incorporating RCA can achieve fresh-state properties comparable to those of conventional SCC [4,12,13,14,15,16].

In the hardened state, the incorporation of RCA typically results in reductions in compressive strength, tensile strength, and modulus of elasticity due to the presence of microcracks and weaker interfacial transition zones associated with the adhered mortar [5,11,17,18,19,20]. Khalaf and DeVenny [14] and Evangelista and de Brito [19] reported compressive strength reductions of approximately 10–25% for full RCA replacement, although the quality and origin of the recycled aggregate play a decisive role. RCA sourced from precast concrete elements has been shown to exhibit superior performance compared to RCA derived from mixed demolition waste [20]. Several authors have also demonstrated that, when high-quality RCA is used, SCC mixtures can satisfy durability requirements related to shrinkage, carbonation, and chloride ingress [8,10].

Karatas & Gunes [21] investigated SCC with polypropylene and steel fiber, noting that fiber addition supports fresh properties and improves mechanical performance, offering promising approaches to counteract RCA-related workability and strength issues, while Abed et al. [22] studied self-compacting high-strength concrete containing RCA, fly ash, cellular concrete, and perlite. They found that RCA at 50% replacement with proper SCMs maintains good mechanical properties and fresh behavior.

While the material behavior of SCC incorporating RCA has been extensively investigated, studies focusing on structural elements remain relatively limited. Existing research has primarily examined simply supported reinforced concrete beams subjected to monotonic loading, where reduced stiffness and cracking load have been reported, but ultimate flexural capacity and ductile failure modes were generally preserved when adequate reinforcement was provided [23,24,25]. Limbachiya et al. [11] observed reduced ultimate strength with increasing RCA content, whereas Padmini et al. [16] reported larger deflections and wider cracks, consistent with the reduced elastic modulus and tensile strength of RAC.

The reinforcement ratio has been identified as a key parameter influencing the structural response of RAC beams. Higher reinforcement ratios can partially compensate for reductions in stiffness and strength associated with RCA, although excessive reinforcement may reduce ductility and promote brittle behavior, particularly in SCC where aggregate interlock is limited [18,19]. Mohammed and Najim [18] confirmed that increased reinforcement improves load-carrying capacity but may negatively affect ductility.

Research on continuous reinforced concrete beams incorporating RCA—particularly those made with SCC—is still scarce. Continuous beams are fundamental structural elements in bridges, buildings, and industrial floors, where both positive and negative bending moments develop over multiple supports. These elements require adequate crack control, ductility, and moment redistribution capacity. Evangelista and de Brito [19] reported only minor reductions in moment redistribution in continuous beams with recycled fine aggregates, while Shawais et al. [24] observed increased crack widths and reduced shear capacity in two-span SCC T-beams with high RCA content, especially in negative moment regions.

Numerical modeling approaches have also been proposed to predict flexural response and crack development in continuous beams with RCA, although accurate modeling of moment redistribution and post-yield behavior remains challenging [25,26]. Recent investigations have expanded the scope to durability and long-term performance. Zheng et al. [27] studied chloride ingress and carbonation resistance in SCC with RCA, reporting that durability losses can be mitigated through proper mix design and SCM incorporation. Skazlić et al. [28] analyzed shrinkage and creep behavior, concluding that RCA slightly increases long-term deformations but remains within acceptable limits for structural applications. 

Despite these advances, experimental data on the flexural behavior of continuous SCC beams incorporating recycled coarse aggregates, particularly considering the combined influence of RCA replacement level and reinforcement ratio, remain limited. Moreover, the interaction between SCC rheology, RCA microstructure, and structural response in negative moment regions has not been sufficiently clarified.

This study addresses these research gaps by conducting an experimental investigation of nine continuous SCC beams, each subjected to two-span loading, with varying reinforcement ratios and RCA replacement levels (0%, 50%, 100%). The aim is to assess the combined effects of recycled aggregate content and reinforcement ratio on load-bearing capacity, cracking behavior, deflection, ductility, and failure modes. In addition, detailed tests on fresh concrete properties (slump flow, T_500_, V-funnel, L-box) and hardened properties (compressive strength, tensile strength, and modulus of elasticity) are performed to support the structural analysis. This dual-level approach contributes to both material science and structural engineering disciplines and offers practical insights for implementing sustainable concrete technologies in load-bearing structural systems.

## 2. Concrete Mixes and Material Properties

### 2.1. Natural and Recycled Aggregates

**Figure 1:**
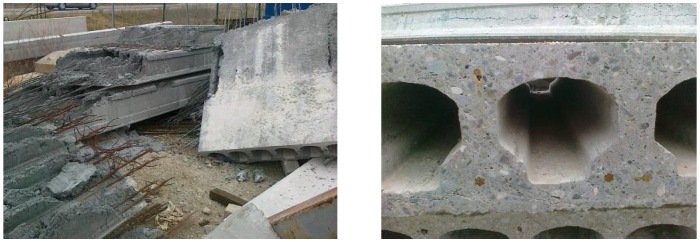
Mechanically damaged hollow-core slabs.

The natural aggregate used in the reference SCC-NAC mix consisted of river aggregate with standard fractions of 0/4 mm and 4/8 mm, and crushed stone aggregate in the 8/16 mm fraction. The recycled aggregate was produced by crushing mechanically damaged hollow-core slabs that had been rejected during production or transport (Figure 1).

These hollow-core slabs were manufactured using extrusion and prestressing technology and were composed of semi-dry concrete with a strength class of C50/60. The mix design included 46% river aggregate (0/4 mm), 32% river aggregate (4/8 mm), and 22% crushed stone aggregate (8/16 mm). The cement content was 450 kg/m^3^, with a water-cement ratio of 0.39.

**Figure 2:**
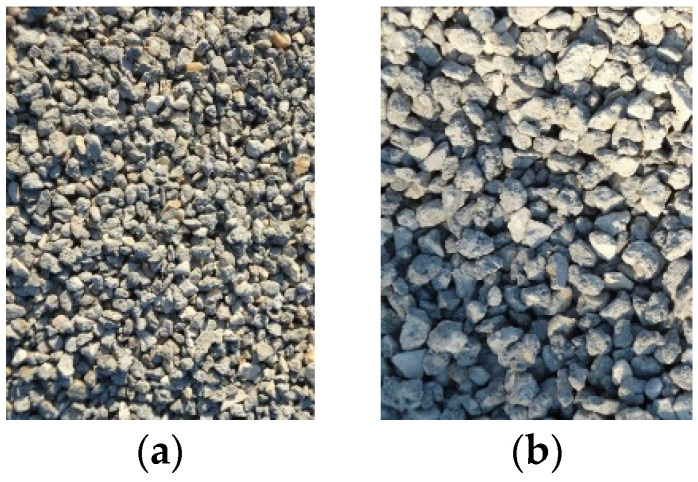
Recycled aggregate: (**a**) 4/8 mm fraction; (**b**) 8/16 mm fraction.

At the time of processing, the hollow-core slabs used for recycling were aged between one and four months. They were crushed to produce high-quality recycled aggregate, which was then separated into two target fractions: 4/8 mm and 8/16 mm (Figure 2).

The recycled aggregate fractions were screened to determine their granulometric (particle size) distribution. Additional tests were carried out to assess the bulk density in both loose and compacted states, as well as the water absorption of each fraction individually (Table 1). These parameters were essential for evaluating the suitability of the recycled aggregates for use in SCC mixtures.

The measured bulk density of the recycled aggregates were slightly lower than those of natural aggregates, which is typically range from 1400 to 1600 kg/m^3^. This reduction reflects the higher porosity and more irregular particle shape of recycled materials. Additionally, the water absorption of recycled aggregates was significantly higher than that of natural aggregates, consistent with their porous microstructure and adhered mortar.

**Figure 3:**
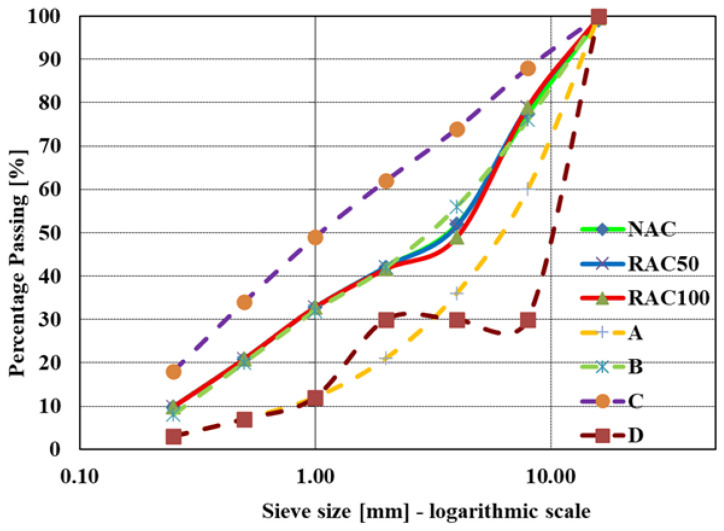
Particle size distribution of aggregate.

For the preparation of the concrete mixtures, the particle size distribution of the aggregates was determined in accordance with SRPS U.M1.057:1984, which defines standard grading curves (Types A–D), as illustrated in Figure 3. This national standard is directly equivalent to the grading methods specified in the European standard EN 933-1:2012 standard [29].

The particle size distributions of the aggregates used in the reference mix with natural aggregate (NAC) and in the mixes with 50% and 100% recycled aggregate replacement (RAC50 and RAC100, respectively) are presented in Figure 3. X-axis represents sieve size in mm, plotted on a logarithmic scale.

The proportions of all other mix components remained constant across all mixtures. However, slight adjustments to the mixing water were necessary to achieve comparable workability, due to the higher water absorption capacity of the recycled aggregates in the RAC50 and RAC100 mixes.

### 2.2. Binder Materials and Concrete Mix Design

The properties of binder materials and the mix proportions for each concrete type, including adjustments for recycled aggregates, are summarized in Table 2.

## 3. Fresh and Hardened Properties of SCC with Natural and Recycled Aggregates

### 3.1. Fresh Concrete Properties

The SCC mixes containing recycled aggregate were designed to achieve the same workability as the SCC mix produced with natural aggregate. The slump flow test for the reference NAC exhibited a spread diameter of 605 mm after lifting the pre-wetted cone, with a T_500_ time of 4.8 s. In comparison, RAC50 and RAC100 mixes achieved spread diameters of 600 mm and 602 mm, respectively, with both mixes showing a T_500_ time of 5.0 s. Based on these results and in accordance with the European Guidelines for Self-Compacting Concrete (EFNARC) [4] and EN 206-9:2010 [30], all SCC mixes, regardless of aggregate type, fall within flow class SF1 (slump flow 550–650 mm), while the recorded T_500_ times correspond to viscosity class VF1 (V-funnel time 4–6 s).

These results indicate that comparable flowability was achieved across all mixes, despite the incorporation of recycled aggregates. According to EFNARC and EN 206-9:2010 the acceptable range for slump flow diameter is 550 to 850 mm. Additionally, in engineering practice, a T_500_ time between 3 and 7 s is generally considered optimal.

### 3.2. Mechanical Properties of Hardened Concrete

**Figure 4:**
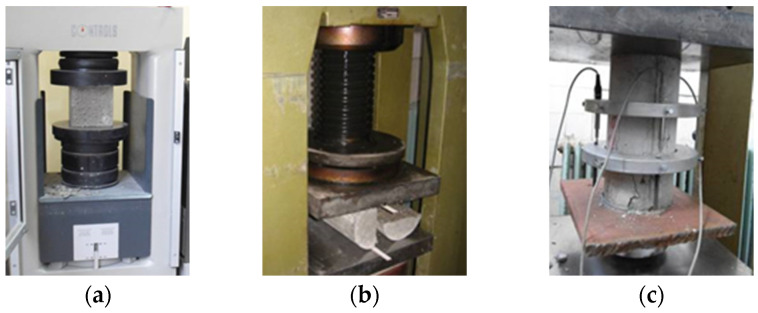
Mechanical Testing of Hardened Concrete: (**a**) compressive strength test, (**b**) tensile splitting test, (**c**) determination of modulus of elasticity.

The hardened concrete was evaluated for compressive strength, splitting tensile strength, and both the tangent and secant moduli of elasticity. Compressive strength tests were conducted on 150 × 150 mm cubes (Figure 4a), in accordance with EN 12390-6 [31], after curing the specimens in water at 20 °C. Testing was carried out at 2, 7, 14, 28, and 365 days.

The splitting tensile strength was determined using cylindrical specimens of Ø150 × 300 mm (Figure 4b), tested at 28 days in accordance with EN 12390-6:2009. Three specimens were tested, and the average value was reported as the final result.

The tangent and secant moduli of elasticity were determined in accordance with EN 1992-1-1:2004 [32], as illustrated in Figure 4c.

The density of the reference concrete (NAC) was 92 kg/m^3^ higher than that of the RAC50 mix, and 49 kg/m^3^ higher than the RAC100 mix, as shown in Table 3. All three mixtures exhibited a pronounced early strength development, achieving more than 60% of their 28-day compressive strength within the first 7 days.

The differences in 28-day compressive strength depend on the composition of the concrete mix. The RAC50 mixture exhibited a 26.85% higher strength, corresponding to an increase of 12.50 MPa compared to the reference NAC. In contrast, the RAC100 mixture showed a reduction of 2.05 MPa, representing a 4.40% decrease compared to NAC. This variation in performance, particularly the significant strength gain observed in RAC50, can be attributed to the microstructural characteristics of concrete incorporating recycled aggregate.

Recycled aggregate differs from natural aggregate in that it comprises two components: the natural aggregate itself and a residual cement paste adhered to its surface. The presence of this adhered paste adversely affects the aggregate’s quality by reducing its density and increasing its water absorption capacity. Literature reports [33,34] indicate that the cement paste content in the 4/8 mm fraction of recycled aggregate ranges from 33% to 55%, whereas in the 8/16 mm fraction it ranges from 23% to 44%. The fine fractions contain the highest proportion of cement paste, which significantly restricts their use in structural concrete.

The mechanical performance of self-compacting concrete (SCC) with recycled aggregate was assessed by comparing experimental results with analytical models widely adopted in design standards [32,35] and prior research [36,37,38,39]. These models define the relationships between compressive strength, tensile strength, and the secant modulus of elasticity. 

The tensile strength of each mixture was analyzed as a function of compressive strength. The experimental results fall within the range predicted by the analytical models of Felekoglu et al. [37] and Kim [39], demonstrating a consistent trend despite the variations in aggregate compositions. Tensile strength values for RAC50 and RAC100 were slightly lower yet comparable to those of NAC, reflecting the influence of recycled aggregate on tensile capacity.

Similarly, the relationship between the secant modulus of elasticity and compressive strength was evaluated. The modulus values for all three concretes closely align with the model proposed by Person [36], with the best fit observed for NAC. The RAC50 and RAC100 mixes show a slight reduction in stiffness, which is expected due to the lower density and higher porosity of recycled aggregates. Nevertheless, the alignment with the reference model confirms that these variations remain within a predictable range.

## 4. Experimental Setup for Structural Testing of Continuous Beams

### 4.1. Beam Geometry and Reinforcement Details

The RC beams tests were conducted on a testing machine in the Mechatronics Laboratory of the Faculty of Mechanical Engineering, University of Niš. The experiments were performed by professional staff from the accredited Laboratory for Testing Structures at the Faculty of Civil Engineering and Architecture, University of Niš, using certified measuring equipment.

**Figure 5:**
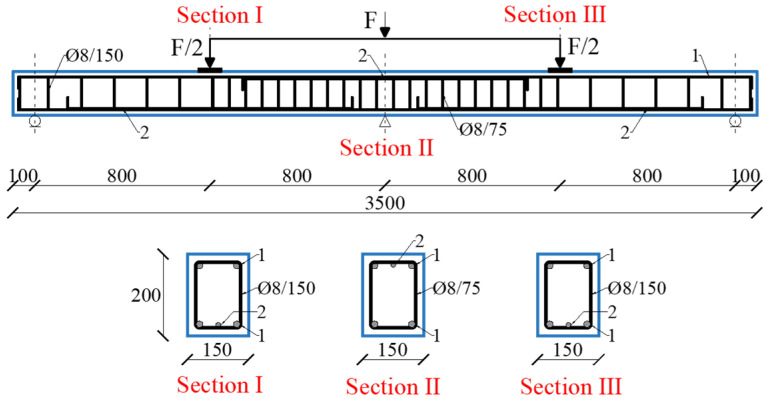
Reinforcement layout of the tested continuous RC beam.

The experimental program comprised nine continuous reinforced concrete (RC) beams with rectangular cross-section measuring 150 mm in width and 200 mm in height (b/h = 150/200 mm). Each beam had a total length of 3400 mm and was designed as a two-span continuous members, with clear span of 1600 mm each (Figure 5). The beams were reinforced with B500B steel (modulus of elasticity: 200 GPa; characteristic yield strength: fy = 500 MPa; ultimate tensile strength: fu = 560 MPa).

For systematic monitoring and comparative analysis, the beams were labeled according to the concrete type and reinforcement ratio used in their construction (Table 4).

### 4.2. Test Setup and Instrumentation

During testing, the continuous beams were loaded using a hydraulic system. The force from the hydraulic piston was applied through two concentrated loads positioned at the midpoints of each span. The load was applied to the concrete beams through 100 mm wide steel plates, with a 30 mm diameter steel roller between them to ensure uniform distribution and permit slight rotation.

Support conditions were simulated using 100 mm wide steel contact plates to represent both fixed and movable supports. Loading was applied in a displacement-controlled mode, with deflection increased at a constant rate of 0.02 mm per second (1.2 mm per min) until beam failure or until no further load increase was observed.

Throughout the experiment, structural responses were recorded, including overall deflections, concrete and steel reinforcement strains, and crack initiation and propagation under increasing load. To facilitate crack observation, the beam sides were marked with a checkerboard pattern starting 50 mm above the bottom edge. Loading was paused at 5.0 kN intervals to manually mark and measure cracks.

Crack development and structural deformation were monitored using electronic and mechanical instrumentation. Crack widths were continuously measured using a dilatometer equipped with Linear Variable Displacement Transducers (LVDTs), all supplied by Hottinger Baldwin Messtechnik (HBM, Darmstadt, Germany), each typically tracking a single crack within its gauge length. To distinguish crack progression at different load levels, individual cracks were marked with color-coded indicators.

Vertical deflections were measured using HBM LVDTs (model W50) at mid-span beneath the applied concentrated load. Strain in the concrete tension zone was monitored with a custom-built dilatometer incorporating W20 type LVDTs with a 100 mm gauge length. This device simultaneously recorded the total crack width over the gauge length within the tensile region of the RC beam.

Strain in the steel reinforcement and the upper surface of the compressed concrete zone was measured using foil-type electrical resistance strain gauges (SGs) (HBM, Darmstadt, Germany) with a nominal resistance of 120 Ω and gauge lengths of 6 mm (reinforcement) and 50 mm (concrete surface). The gauges, were bonded to the steel using Z70 adhesive (HBM) prior to casting, enabling their embedding with the reinforcement bars. Protective coatings were applied to steel-mounted gauges to prevent environmental damage. On the concrete surface, gauges were affixed using a two-component adhesive (X60, HBM, Darmstadt, Germany), without additional protection. Placement on the reinforcement was determined from predicted stress distributions, targeting zones of maximum expected tension in both spans and support regions.

To supplement the electronic instrumentation, mechanical devices were also used for displacement and crack width measurements. Crack widths were additionally measured using a ZDI–VDA gauge with a resolution of 0.05 mm. Detailed visual inspection was carried out using a magnifying glass and an optical microscope, both capable of detecting changes as small as 0.05 mm.

**Figure 6:**
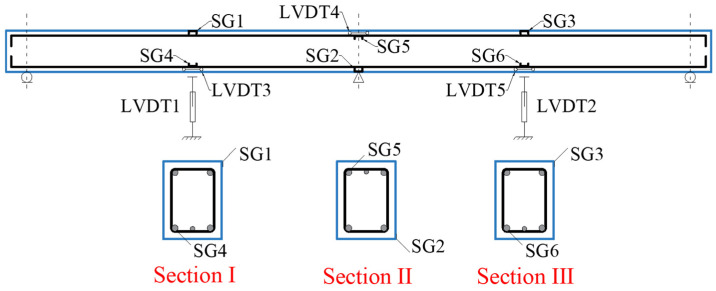
Positions of SGs and LVDTs in critical cross-sections of the tested beams.

The spatial arrangement of the strain gauge tapes (SG) and Linear Variable Displacement Transducers (LVDTs) across the instrumented sections of the tested beams is summarized in Figure 6. Sections I, II, and III correspond to critical cross-sections selected based on anticipated stress distributions, including mid-span and support zones.

The applied load was measured using an electronic dynamometer (model U2A, HBM, Darmstadt, Germany) with a maximum capacity of 100 kN and an accuracy of ±0.5%. Signal acquisition and data logging were performed using data acquisition systems manufactured by HBM, including the MGCplus and SPIDER8 units. These systems interfaced with a personal computer, enabling automatic measurement at one-second intervals (quasi-dynamic mode), with calibration, real-time monitoring, and data processing managed using the Catman 5.1 software suite (HBM).

## 5. Structural Performance of Continuous Beams: Results and Discussion

Literature about continuous beams made of self-compacting or recycled aggregate concrete remains scarce. Recent studies such as Shawais et al. [24] on hybrid SCC T-beams with varying RCA content, and Alkhteeb and Dawood [40] on continuous RAC beams, contribute to expanding this limited dataset.

A comparative analysis of the experimental results was conducted to evaluate the influence of concrete type on the structural performance of continuous beams. For specimens with identical reinforcement ratios, the measured mid-span deflections and longitudinal dilatations were systematically compared according to the concrete mixture employed.

### 5.1. Load–Deflection Behavior

#### 5.1.1. Experimental Load–Deflection Results

Special attention was devoted to the analysis of load–deflection behavior, as it provides a direct measure of the structural response under applied loads. The deflection characteristics of continuous beams cast with RAC were compared to those of beams made with NAC, maintaining identical reinforcement ratios at mid-span Sections I and III.

Beams with recycled aggregates exhibited comparable ultimate load capacity as those with natural aggregates, particularly at lower replacement levels. This observation is consistent with findings of Martinez et al. [41] on reinforced concrete beams made with high-percentage coated recycled aggregates, which reported only modest reductions in flexural capacity despite significant aggregate replacement.

The load-deflection curves exhibited three distinct stages during loading: (1) an initial elastic phase up to the formation of the first cracks, (2) a nonlinear phase between cracking and yielding of the tensile reinforcement, and (3) a post-yield phase leading ultimately to failure. For all tested specimens, the elastic region under low loads levels displayed almost identical deflection responses prior to crack initiation. Once cracking occurred, the load-deflection relationship transitioned into a distinctly nonlinear regime.

**Figure 7:**
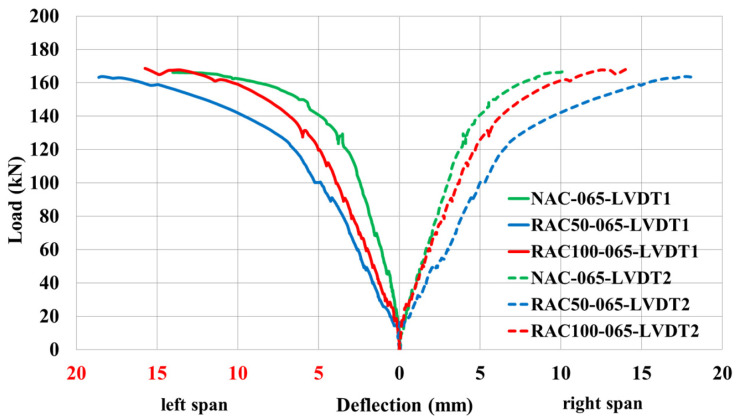
Load–deflection response at Section I and Section III for beams with a reinforcement ratio of ρ = 0.65%.

Figure 7 presents the load–deflection responses Sections I and III for beams NAC-065, RAC50-065, and RAC100-065 under progressively increasing load levels. The results indicate that beams incorporating recycled aggregates (RAC50-065 and RAC100-065) exhibited larger deflections than the NAC-065 beam at identical load levels. Notably, the deflection difference between NAC-065 and RAC100-065 was smaller than that between NAC-065 and RAC50-065 under equivalent loading conditions.

**Figure 8:**
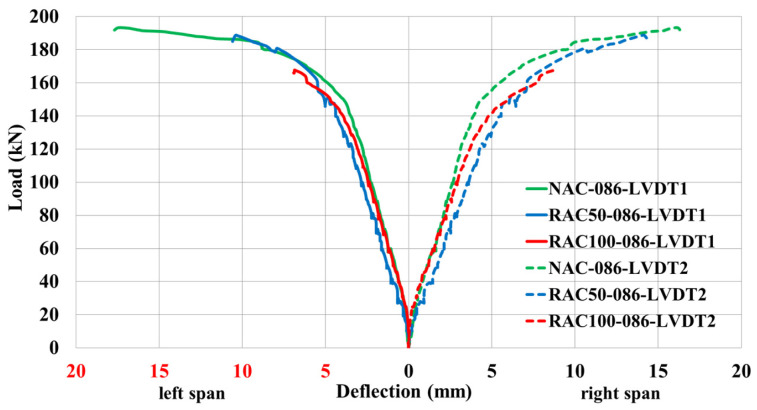
Load–deflection response at Section I and Section III for beams with a reinforcement ratio of ρ = 0.86%.

Figure 8 illustrates that the ultimate load capacity of the beam reinforced at 0.86% with 100% recycled aggregate (RAC100-086) is significantly lower than that of the corresponding beams containing 50% recycled aggregate (RAC50-086) and natural aggregate (NAC-086). It is noteworthy that up to the yielding of the tensile reinforcement, all beams (NAC-086, RAC50-086, RAC100-086) exhibited nearly identical deflection trends.

**Figure 9:**
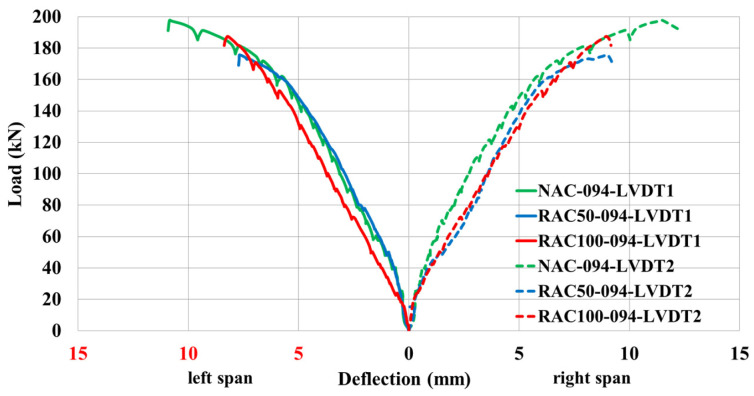
Load–deflection response at Section I and Section III for beams with a reinforcement ratio of ρ = 0.94%.

According to Figure 9, beams incorporating recycled aggregates (RAC50-094, RAC100-094) exhibited lower ultimate load capacities than the beam cast with natural aggregate concrete (NAC-094).

#### 5.1.2. Ductility, Serviceability, and Code-Based Comparisons

The ductility of the tested beams was evaluated using displacement-based indices derived from the load–deflection responses. The ductility indices (*μ*) were calculated as the ratio of the displacement at maximum load to the displacement at yielding of the tensile reinforcement, following a commonly used experimental approach (Table 5). The resulting values fall within ranges typically considered satisfactory, ranging from approximately 3 to 6, and are consistent with tension-controlled behavior criteria suggested in ACI 318 and reinforcement classes recommended in EC2, as well as with the methodology of Park and Paulay [42].

The ductility indices indicate that all beams maintained sufficient post-yield deformation to allow redistribution of internal forces, consistent with continuous beam behavior, while exhibiting variations according to reinforcement ratio and concrete type.

Serviceability behavior was assessed by considering beam displacements under a load corresponding to approximately 50% of the ultimate load (0.5F_u_) (Table 6). In structural concrete literature, service load levels are often expressed as a proportion of the ultimate capacity. Bischoff [43] indicates that serviceability checks for steel-reinforced concrete elements typically correspond to about 60–65% of the nominal (ultimate) moment capacity, which implies that service loads can be represented as approximately (0.4–0.65)F_u_ for practical evaluation of deflections and cracking behavior. Therefore, in this study a service load level of ~0.5F_u_ was chosen to assess experimental deflections and to compare them against Eurocode and ACI serviceability criteria.

For the tested span of 1600 mm, the observed mid-span deflections at the service load of ~0.5F_u_ remain well within code-specified limits, corresponding to δ/L ≤ 1/250 (6.40 mm) according to EC2, and δ/L ≤ 1/360 (4.44 mm) according to ACI 318. These results indicate that all beams satisfy standard serviceability requirements.

The observed deflections at this load level demonstrate that all beams satisfy typical serviceability requirements. The measured mid-span deflections at ~0.5F_u_ vary according to concrete type and reinforcement ratio. Some RAC beams exhibit higher deflections than their NAC counterparts, while others show comparable or slightly lower values, reflecting the combined influence of concrete stiffness and reinforcement configuration.

The ultimate moments of each span were calculated according to EC2 and ACI 318 provisions, providing a code-based reference for comparison (Table 7). The ultimate limit load of each span in the continuous beams was estimated using the standard relation F_u_/2 = 6M_u_/L where M_u_ is the ultimate moment of the cross-section and L is the span length. This approach is grounded in plastic analysis of continuous beams, in which each span is considered as a single span between fully developed plastic hinges at the supports.

The comparison indicates that the code-based predictions provide conservative estimates of the ultimate loads for all specimens. Experimental ultimate loads often exceed the theoretical 6M_u_/L values due to moment redistribution across spans, enabled by the sufficient ductility of the reinforcement and the ability of the concrete sections to develop plastic hinges, as observed in the tested beams. This confirms that the applied simplifications offer a practical and reliable estimate for continuous SCC–RAC beams.

#### 5.1.3. Discussion on Reinforcement Ratio and Concrete Type Effects

In summary, beams cast with self-compacting concrete containing high-quality recycled aggregate (RAC50 and RAC100) consistently exhibited greater deflections than their natural aggregate counterparts. This behavior is primarily attributed to differences in the tensile zone response associated with variations in aggregate composition. Since the elastic moduli and cracking patterns are comparable across all concrete types, the observed variations in deflection can be primarily attributed to the interaction of the tensioned concrete between adjacent cracks. Similar tendencies were reported by structural tests on continuous RAC beams by Alkhteeb and Dawood [40], where ultimate loads decreased up to ~5% with increased RCA content, albeit with somewhat higher deflections.

The experimental results demonstrated a clear interaction between reinforcement ratio and concrete type in influencing the structural behavior of SCC beams. In the group with the lowest reinforcement ratio (ρ = 0.64%), notable differences were observed in the load-deflection response, cracking behavior, and ultimate capacity among beams incorporating NAC, 50% RAC, and 100% RAC. Beams cast with 100% RAC exhibited lower stiffness and earlier cracking initiation compared to those made with NAC, reflecting the reduced mechanical properties of RAC reported in previous studies [5]. However, as the reinforcement ratio increased to 0.85% and 0.94%, the differences among concrete types became less pronounced. At these higher reinforcement levels, the load-bearing behavior was predominantly governed by the steel reinforcement, resulting in enhanced stiffness and ductility regardless of the concrete mixture. This observation aligns with established findings indicating that, at higher reinforcement ratios, the overall structural response is increasingly controlled by the steel contribution [35]. Furthermore, deflection profiles of heavily reinforced beams were largely comparable, suggesting that increased steel content mitigates the adverse effects associated with RAC’s lower mechanical quality. Collectively, these results underscore the critical role of reinforcement ratio in evaluating the structural applicability of recycled aggregate concrete.

### 5.2. Steel and Concrete Strains

**Figure 10:**
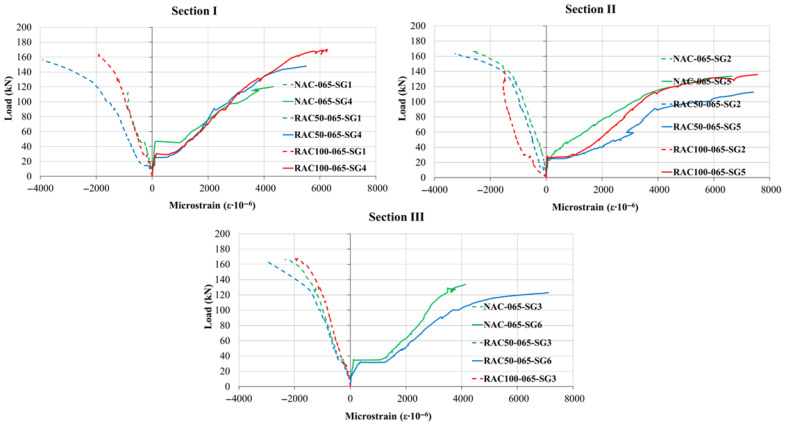
Load–Strain Curves at Sections I, II, and III for beams with a reinforcement ratio of ρ = 0.65%.

**Figure 11:**
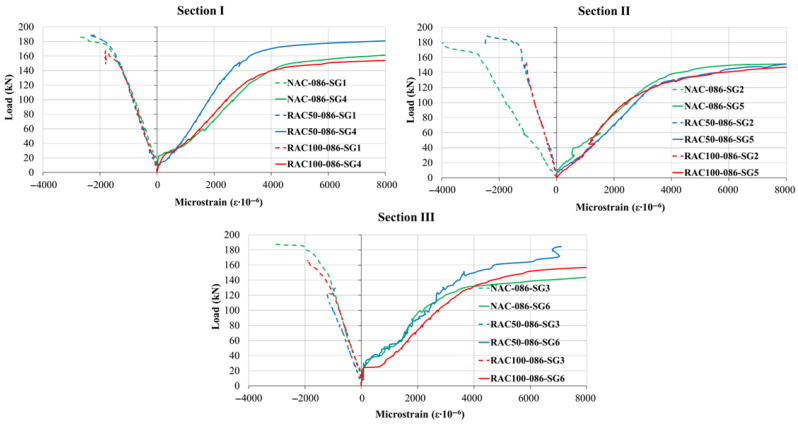
Load–Strain Curves at Sections I, II, and III for beams with a reinforcement ratio of ρ = 0.86%.

**Figure 12:**
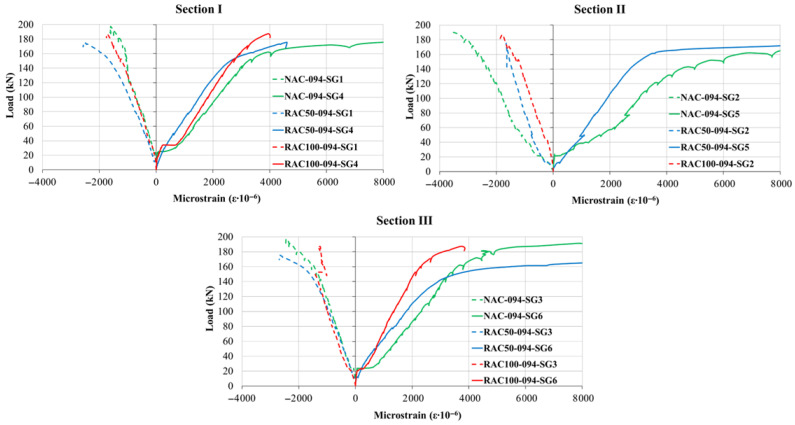
Load–Strain Curves at Sections I, II, and III for beams with a reinforcement ratio of ρ = 0.94%.

The overall shape of the load–strain curves was comparable across all the aggregate type, indicating that the substitution of natural aggregate with recycled aggregate did not significantly modify the qualitative response of the beams. However, the nominal strain magnitudes varied depending on both the reinforcement ratio and the aggregate type, as illustrated in Figure 10, Figure 11 and Figure 12. The measured tensile-zone strains comprised contributions from concrete elongation (approximately 2%) and crack formation (approximately 98%), implying that the total elongation can be interpreted predominantly as the cumulative crack width over the gauge length. Because a single crack generally developed within the 100 mm measurement base, the corresponding crack width could be determined as a continuous function of the applied load with high precision. Once cracking occurred, tensile stresses were carried almost entirely by the reinforcement; therefore, the recorded strains can be regarded as representative of the steel strain at an equivalent distance from the neutral axis.

The load–strain relationship can generally be divided into three distinct stages. The first stage is linear and extends up to the initiation of the first crack in the tensile zone. The onset of cracking marks the beginning of the second stage, characterized by a pronounced reduction in stiffness and a nonlinear increase in strain up to the yielding of the tensile reinforcement. The third stage begins after steel yielding, during which strain increases rapidly with relatively small increments in load, exhibiting strong nonlinearity until failure. In this phase, stress redistribution occurs from the concrete to the reinforcement as a result of bond degradation and crack propagation.

For all reinforcement ratios, beams incorporating recycled aggregate (RAC50 and RAC100) exhibited slightly higher strain values compared to those made with natural aggregate, particularly in the compressive zones under low load levels. This behavior suggests that SCC containing recycled aggregate tends to display a more pronounced nonlinear response even during the early stages of loading. Nevertheless, the overall deformation pattern and the sequence of behavioral phases remained comparable among all beam types.

In the compressive zones, concrete strains were continuously recorded throughout the loading using strain gauges affixed to the upper fiber of the cross-section, whereas in the tensile zones, measurements were obtained near the bottom fiber of the cross-section using LVDT sensors. The load–strain response in compression exhibited a trend similar to that in the reinforcement, with slightly reduced stiffness observed in beams incorporating recycled aggregate. These results indicate that although the use of recycled aggregate marginally increases deformation and decreases stiffness, the fundamental mechanical response and crack formation mechanism remain consistent with those of beams made cast natural aggregate concrete.

These findings confirm that the incorporation of recycled aggregate in self-compacting concrete beams primarily influences the quantitative aspects of strain development, while preserving comparable overall flexural behavior and ductility to those beams made with natural aggregate. This demonstrates the potential of recycled aggregate concrete as a viable material for sustainable structural applications. Moreover, a recent long-term numerical and experimental study by [44] suggests that recycled-aggregate concrete beams maintain acceptable deflection and crack propagation behavior under sustained loading, which supports the structural viability of RAC beyond short-term static tests.

### 5.3. Crack Formation, Development, and Width

The formation and propagation of cracks in the tested continuous self-compacting concrete (SCC) beams were monitored visually along pre-marked lateral surfaces. Cracks patterns were traced using color codes corresponding to the load levels at which each crack first appeared, enabling clear identification of initiation and subsequent development. This distribution of cracks was analyzed in relation to different load levels, reinforcing ratios, and concrete types, providing a detailed picture of how cracks evolved and where they were most prominent. In parallel with visual inspection, crack widths were continuously measured using displacement transducers installed at midspan of the first span and above the central support. Measurements were recorded at one-second intervals, allowing high-resolution tracking of crack width evolution throughout the loading process.

**Figure 13:**
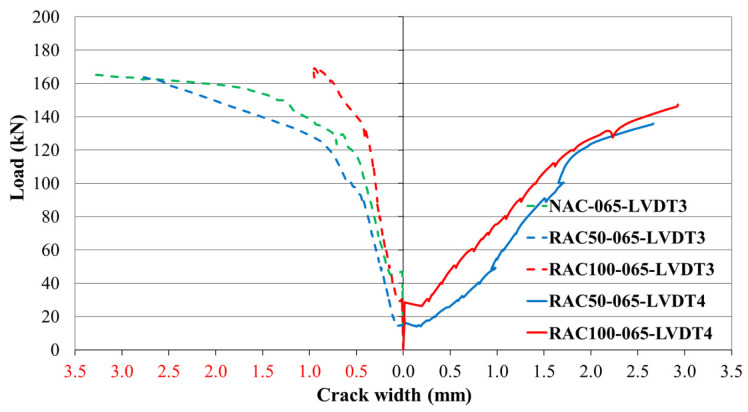
Load–crack relationship for beams with a reinforcement ratio of ρ = 0.65%.

**Figure 14:**
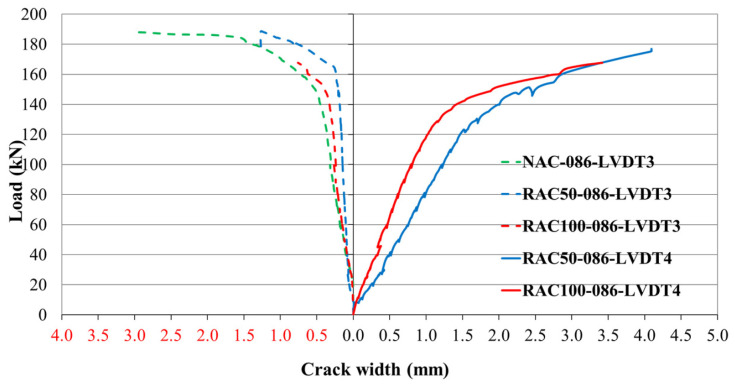
Load–crack relationship for beams with a reinforcement ratio of ρ = 0.86%.

**Figure 15:**
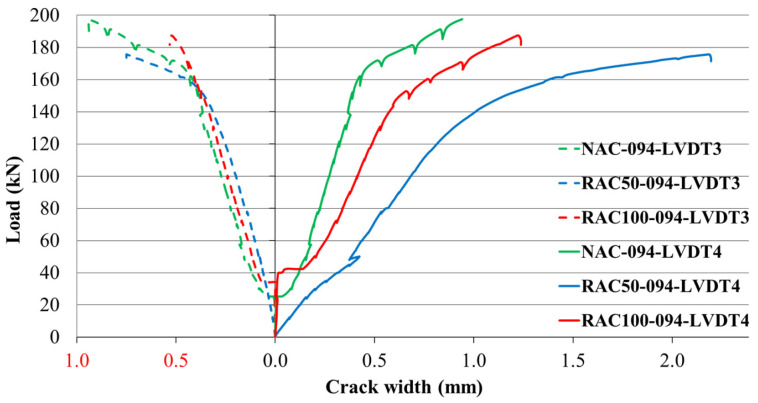
Load–crack relationship for beams with a reinforcement ratio of ρ = 0.94%.

At low load levels, strain increments in the concrete remained negligible prior to the onset of cracking. As the applied load increased, additional cracks formed progressively. Once reinforcement yielding began, the rate of crack initiation accelerated and the widths of existing cracks increased rapidly within the plasticized regions of the reinforcement. The distribution of these cracks was influenced by both the applied load and the concrete composition, with cracks forming at different locations depending on the concrete type and reinforcement ratio. Figure 13, Figure 14 and Figure 15 illustrate the relationships between the applied load and the corresponding crack widths for beams produced with different SCC mixtures and reinforcement ratios. The results show that the load level at which the first cracks formed was influenced by both the concrete type and the reinforcement ratio.

In beams with a reinforcement ratio of ρ = 0.65%, cracks developed almost simultaneously within the span and above the central support, although the load level required to initiate cracking varied depending on the concrete composition (Figure 13).

In beams with ρ = 0.86%, those cast with recycled aggregate concrete (RAC) exhibited the first cracks above the middle support at relatively low load levels, after which cracking progressed into the spans. In contrast, beams made with natural aggregate concrete (NAC) tended to develop their initial cracks within the span region. For beams produced entirely with recycled aggregate (RAC100), cracking initiated predominantly in the spans, whereas cracks above the support formed at later stages or outside the instrumented region (Figure 14).

In beams with ρ = 0.94%, the initiation of the first crack was strongly dependent on the concrete type. In NAC beams, initial cracks developed almost simultaneously in the span and above the central support. In RAC50 beams, cracking initiated above the support at relatively low load levels, whereas in RAC100 beams, the first cracks appeared predominantly in the span region (Figure 15).

**Figure 16:**
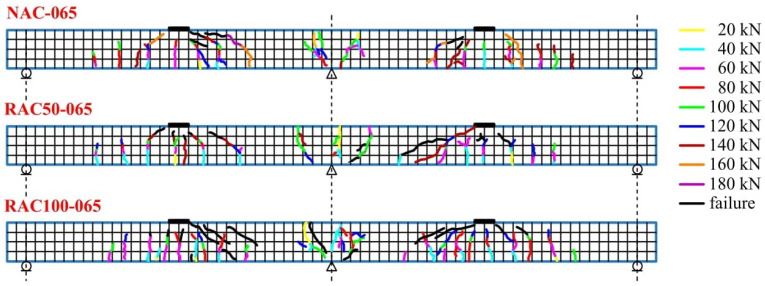
Crack pattern maps for beams with a reinforcement ratio of ρ = 0.65%.

**Figure 17:**
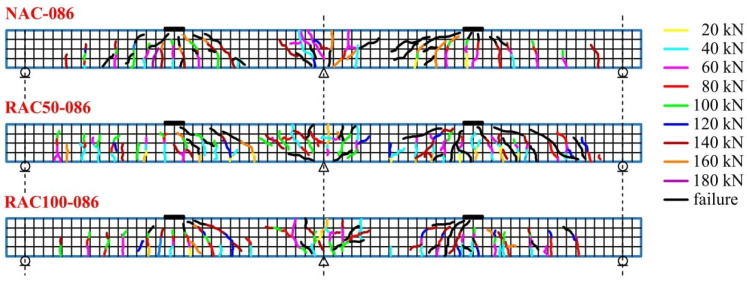
Crack pattern maps for beams with a reinforcement ratio of ρ = 0.86%.

**Figure 18:**
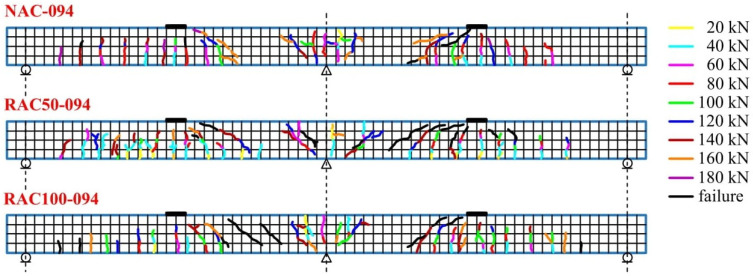
Crack pattern maps for beams with a reinforcement ratio of ρ = 0.94%.

The crack maps shown in Figure 16, Figure 17 and Figure 18 provide a visual representation of crack progression and distribution of cracks at various load levels. These figures detail how cracks evolved at specific points on the beam, with a focus on the locations and directions of crack formation relative to applied loads. Vertical cracks were observed directly above the central support and beneath the applied loads, whereas diagonal cracks (approximately 45°) formed between the load application points and the central support. Between the outer supports and the loading points, cracks were predominantly vertical. 

The average crack spacing and the crack lengths were found to depend primarily on the reinforcement ratio, with the influence of the concrete type was comparatively less significant. The maps of crack distribution for beams with a reinforcement ratio of ρ = 0.65%, ρ = 0.86%, and ρ = 0.94% (Figure 16, Figure 17 and Figure 18) reveal important insights into the correlation between reinforcement, crack development, and load levels. As the applied load increased, the number and width of the cracks also expanded, with variations observed between different reinforcement ratios.

For beams made with SCC incorporating recycled aggregates, crack initiation occurred at lower load levels compared to those made with natural aggregate SCC. In particular, beams with recycled aggregates exhibited larger maximum crack widths at failure, indicating greater crack opening associated with the use of recycled aggregates. This is reflected in the visual data provided in Figure 16, Figure 17 and Figure 18, where cracks formed at different points depending on the type of concrete.

Figure 16, Figure 17 and Figure 18 show the progressive formation of cracks at various stages of loading, with different crack patterns observed depending on the type of concrete. The relationship between the applied load and crack width for beams produced with different concrete mixtures and reinforcement ratios is clearly illustrated in these figures, emphasizing the variations in crack distribution.

Similar tendencies regarding the influence of material composition and internal structural changes on crack initiation and width were reported by Jafari et al. [45], highlighting the sensitivity of SCC beam performance to variations in material conditioning and internal reinforcement.

## 6. Conclusions

The performance of recycled aggregate concrete (RAC) is closely associated with the quality and composition of the recycled constituents. This study demonstrates that high-quality recycled coarse aggregates from precast elements can be successfully incorporated into self-compacting concrete (SCC) without compromising mechanical performance. Essential physical properties—particularly density and water absorption—must be assessed to ensure reliable mix design and consistent mechanical behavior.

Owing to the higher water absorption of recycled aggregates, adjustments to the mix proportions were required to maintain uniform workability. All SCC mixes maintained satisfactory fresh-state properties, confirming the effectiveness of the mix design and suitability for structural applications.

Mechanical testing showed that RAC50 achieved a 26.85% increase in compressive strength with the control mix, whereas RAC100 exhibited only a slight reduction (4.4%). Splitting tensile strength and modulus of elasticity were either slightly enhanced or only modestly affected, demonstrating that partial replacement with high-quality recycled aggregates can provide structural benefits.

These findings highlight the potential of selective recycling as a sustainable approach, offering both environmental and technical advantages in structural concrete design.

Differences in structural behavior among beams with NAC, 50% RAC, and 100% RAC were most pronounced in the group with the lowest reinforcement ratio (ρ = 0.64%). As the reinforcement ratio increased to 0.85% and 0.94%, the influence of the concrete types diminished. This trend indicates that, at higher reinforcement ratios, beam behavior is increasingly governed by the steel reinforcement, thereby reducing the relative contribution of the concrete properties.

A key factor underlying the favorable performance of RAC in this study is the high quality of the source material used to produce the recycled aggregates. In contrast to many experimental programs that rely on demolition waste or concrete of unknown or low strength, the recycled aggregates in this investigation were obtained from precast slabs originally manufactured using high-strength, quality-controlled concrete. This origin directly contributed to the improved mechanical performance of RAC, particularly in terms of stiffness, strength, and deformation behavior under load. Previous studies have demonstrated that the quality of the parent concrete strongly influences the properties of RCA and, consequently, the mechanical response of RAC. When the parent concrete is dense, durable, and of high strength, the attached mortar on the recycled aggregate exhibits superior quality, leading to reduced degradation of key mechanical properties such as compressive strength and modulus of elasticity. The present findings corroborate this understanding, as RAC beams, especially those incorporating partial replacement, displayed structural behavior comparable to that of NAC beams when appropriately reinforced. These results highlight the importance of selective demolition, controlled source separation, and material traceability as effective strategies for enhancing the structural reliability of RAC in practical applications, particularly in scenarios where structural where performance consistency is critical.

The observed mid-span deflections under service-level loads (~0.5F_u_) were within code-specified limits for all beams, indicating satisfactory serviceability behavior for RAC beams.

It should be emphasized that each beam configuration investigated in this experimental program was represented by a single specimen. Consequently, the inherent variability associated with concrete heterogeneity, cracking processes, and local material irregularities could not be statistically quantified. The observed trends in cracking load, stiffness, and ultimate capacity—particularly the enhanced performance recorded for the RAC50 beams—should therefore be interpreted as indicative rather than statistically conclusive.

Moreover, the applicability of the present results is closely linked to the high quality of the recycled coarse aggregates used in this study, which were sourced from controlled, high-strength precast concrete elements. The findings should not be indiscriminately generalized to recycled aggregates originating from mixed demolition waste or from parent concrete of lower or unknown strength. Future research should extend the present work by considering recycled aggregates from mixed demolition sources, parent concretes of varying strength levels, and multiple specimens per configuration to enable a statistical assessment of experimental variability.

Future studies should also investigate the long-term structural performance of RAC under sustained loading, freeze–thaw cycles, or chloride exposure prior to structural testing, to better understand durability-related cracking, stiffness evolution, and deformation behavior.

## Figures and Tables

**Table 1:** Recycled Aggregate Properties.

Fraction	Bulk Density (Loose) [kg/m^3^]	Bulk Density (Compacted) [kg/m^3^]	Water Absorption [%]
4/8 mm	1196	1306	4.13
8/16 mm	1215	1300	4.08

**Table 2:** Composition and material properties of the concrete mixtures.

Component	Description/Properties	Quantity/Dosage
Cement (CEM I 42.5 R, HOLCIM)	Specific gravity: 3.13 g/cm^3^ Setting time: 176–226 min Bulk density: 930 kg/m^3^ (loose), 1515 kg/m^3^ (compacted) Compressive strength: 31.33 MPa (2 d), 55.15 MPa (28 d) Flexural strength: 6.98 MPa (2 d), 9.30 MPa (28 d)	430 kg/m^3^
Filler (Limestone stone flour)	Specific mass: 2.692 g/cm^3^ Void fraction (Rigden): 25.4%	100 kg/m^3^
Admixture (MC PowerFlow 1102)	Polycarboxylate-based high-range water reducer	0.5% by weight of binder
Sand (0–4 mm)	Natural sand	807.5 kg/m^3^
Coarse Aggregate 4–8 mm	Natural or recycled, depending on mix	380 kg/m^3^
Coarse Aggregate 8–16 mm	Natural or recycled, depending on mix	553 kg/m^3^
Water	Adjusted for recycled aggregate absorption	210.0 kg/m^3^ (NAC) 215.0 kg/m^3^ (RAC50) 219.3 kg/m^3^ (RAC100)

**Table 3:** Measured Mechanical Properties of Hardened Concrete.

**Compressive Strength (MPa)**					
	f_c,2_	f_c,7_	f_c,14_	f_c,28_	f_c,365_
NAC	20.40	30.75	42.45	46.55	57.65
σ	1.07	2.07	2.36	0.92	3.45
RAC50	30.15	50.65	60.25	59.05	59.70
σ	0.28	1.91	0.55	5.21	3.30
RAC100	14.65	28.90	39.15	44.50	56.05
σ	0.17	0.93	2.95	0.50	3.61
**Tensile Strength (MPa)**					
	NAC		RAC50		RAC100
f_ct,28_	3.80		4.00		4.50
σ	0.09		0.06		0.13
**Modulus of Elasticity (GPa)**					
	NAC		RAC50		RAC100
E_c_	26.12		27.98		24.86
σ	0.86		3.41		3.94
E_cm_	25.55		25.22		24.35
σ	4.44		0.16		1.63
**Density (kg/m^3^)**					
NAC		RAC50		RAC100	
2325		2233		2276	

σ denotes standard deviation.

**Table 4:** Longitudinal reinforcement and reinforcement ratios (ρ) of tested beams.

Beam ID	Main Reinforcement	Additional Reinforcement	Reinforcement Ratio ρ (%)
NAC-065	2Ø10	–	0.65
NAC-086	2Ø10	1Ø8	0.86
NAC-094	2Ø12	–	0.94
RAC50-065	2Ø10	–	0.65
RAC50-086	2Ø10	1Ø8	0.86
RAC50-094	2Ø12	–	0.94
RAC100-065	2Ø10	–	0.65
RAC100-086	2Ø10	1Ø8	0.86
RAC100-094	2Ø12	–	0.94

**Table 5:** Calculated displacement-based ductility indices (*μ*) for tested continuous beams.

Beam ID	*μ*	Beam ID	*μ*	Beam ID	*μ*
NAC-065	7.40	NAC-086	6.30	NAC-094	4.95
RAC50-065	5.40	RAC50-086	5.70	RAC50-094	4.40
RAC100-065	5.30	RAC100-086	4.00	RAC100-094	4.20

**Table 6:** Mid-span deflections δ (0.5F_u_) of tested continuous beams under a service load of approximately 0.5F_u_.

Beam ID	δ (0.5F_u_) [mm]	Beam ID	δ (0.5F_u_) [mm]	Beam ID	δ (0.5F_u_) [mm]
NAC-065	2.17	NAC-086	2.32	NAC-094	2.80
RAC50-065	3.77	RAC50-086	2.95	RAC50-094	2.76
RAC100-065	2.99	RAC100-086	2.07	RAC100-094	3.30

**Table 7:** Comparison of calculated ultimate moments according to EC2 and ACI 318, theoretical limit loads, and experimentally measured ultimate loads for tested beams.

Beam ID	M_u_ (EC2) [kNm]	F_u_ (EC2) [kN]	M_u_ (ACI 318) [kNm]	F_u_ (ACI 318) [kN]	F_u_ (exp.) [kN]
NAC-065	15.15	113.62	15.11	113.32	166.26
NAC-086	19.52	146.40	19.67	147.52	191.95
NAC-094	21.57	161.77	21.38	160.35	193.56
RAC50-065	15.15	113.62	15.11	113.32	163.35
RAC50-086	19.62	147.15	19.67	147.52	184.92
RAC50-094	21.73	162.97	21.38	160.35	169.02
RAC100-065	15.15	113.62	15.11	113.32	168.66
RAC100-086	19.68	147.60	19.67	147.52	165.96
RAC100-094	21.60	162.00	21.38	160.35	181.59

## Data Availability

The original contributions presented in this study are included in the article. Further inquiries can be directed to the corresponding author.
